# Editorial: Hippocampal function and reinforcement learning

**DOI:** 10.3389/fncom.2025.1595369

**Published:** 2025-04-08

**Authors:** Arij Daou, Hyunsu Lee

**Affiliations:** ^1^Neurophysiology and Computational Neuroscience Group, Biomedical Engineering Program, American University of Beirut, Beirut, Lebanon; ^2^Department of Physiology, School of Medicine, Pusan National University, Yangsan, Republic of Korea; ^3^Research Institute for Convergence of Biomedical Science and Technology, Pusan National University Yangsan Hospital, Yangsan, Republic of Korea

**Keywords:** hippocampus, reinforcement learning, memory consolidation, contextual representation, neural replay, neuromodulation

The hippocampus has historically been recognized as a critical neural substrate for the formation and retrieval of episodic memories. Concurrently, reinforcement learning (RL) has emerged as a foundational framework in both psychology and artificial intelligence for understanding how organisms and computational agents optimize behavior through experience. This Research Topic synthesizes these traditionally separate domains, revealing how hippocampal processes not only support memory functions but also fundamentally influence adaptive decision-making guided by reward and punishment signals.

A central theme unifying the contributions in this Research Topic is the recognition that hippocampal circuits do not merely store static representations of past experiences. Rather, they continuously integrate reward predictions, contextual information, and future-oriented simulations into dynamic memory representations. This integrative processing suggests that hippocampal-dependent memory consolidation and reinforcement-based learning strategies are deeply interconnected systems that mutually shape cognitive processes across multiple timescales.

Tesler et al. tackle the intricacies of scaling from local synaptic processes to large-scale oscillatory phenomena relevant to learning. Their multiscale model of the CA1 region underscores why bridging cellular mechanisms with broader network activity is critical for simulating hippocampal contributions to brain-wide RL tasks.

The notion that reinforcement signals can drive persistent adaptation is explored by Yoder et al., who demonstrate how biologically inspired RL feedback shapes central pattern generation within recurrent neural networks. By illustrating how neuromodulatory signals guide network plasticity over the lifespan, their study illuminates how hippocampal and related circuits maintain the capacity for behavioral recalibration.

In considering offline replay and consolidation, Lee and Jung propose that hippocampal replay events parallel the “Dyna” RL approach. Their framework posits that CA3 simulates multiple scenarios while CA1 evaluates each for potential reward, thereby selectively strengthening connections tied to high-value outcomes. This reframes replay from a purely episodic recapitulation to a computational mechanism for optimizing future performance.

Adaptation to contextual cues takes center stage in Kappel and Cheng. Demonstrating that global remapping of place cell firing patterns facilitates the renewal of extinguished behaviors, they show that hippocampal circuits dynamically re-encode changing environments, allowing organisms to flexibly re-engage learned associations when contexts shift.

Building on the idea of mental simulation, Kim and Lee present “Meta-Dyna,” a computational model that unites hippocampal replay with prefrontal meta-control. Hippocampal simulations rehearse potential outcomes, while prefrontal circuits arbitrate which simulations to adopt–thus enabling swift adaptation under dynamic or uncertain conditions. This synthesis of biologically inspired loops underscores how hippocampal and frontal circuits collaborate to bolster RL processes.

Methodological advances in reinforcement-driven decoding are highlighted by Zhang et al., who describe an EEG-based attention model. While primarily centered on neural data analysis, the selective focus on reward-contingent signals echoes hippocampal processes, where significant stimuli and memories receive enhanced representation. This underscores the translational potential of RL in parsing real-time neural recordings for clinical or research applications.

Finally, a comparative perspective is offered by Lochner et al., who apply RL principles to insect navigation in mushroom body–like circuits. The authors highlight parallels to hippocampal map-based strategies for spatial representation, suggesting a broad, cross-species principle where neural substrates fuse reward information with spatial cues to optimize navigational behavior.

The studies in this Research Topic converge on several key themes that advance our understanding of hippocampal function in reinforcement-based learning. First is contextual flexibility, whereby hippocampal processing encodes environmental transitions and enables rapid retrieval of prior learning when contextual cues reemerge. Another prominent theme is offline replay and consolidation: through simulating possible scenarios, the hippocampus refines subsequent behavior, consistent with RL principles of planning and exploration. In addition, these articles highlight biologically plausible RL, revealing how neuromodulatory feedback in recurrent networks and mental simulations in prefrontal-hippocampal circuits can implement or inspire novel RL algorithms. Lastly, adaptive navigation emerges as a unifying aspect across species and neural systems, demonstrating how spatial representations informed by reinforcement signals guide goal-directed movement and decision-making.

These insights carry significant implications for clinical contexts. Conditions that impair hippocampal function—such as Alzheimer's disease, cognitive aging, or post-traumatic stress disorder—often disrupt both reward-based learning and contextual processing. Leveraging RL-based computational models to explore hippocampal plasticity may inform targeted therapies, from pharmacological interventions enhancing beneficial plasticity to cognitive rehabilitation exercises anchored in reward-driven strategies.

By integrating theoretical perspectives, experimental investigations, and computational modeling, this Research Topic reframes the hippocampus as not merely a depot for episodic memories but an active driver of reinforcement-based decision-making and cognitive flexibility. Future research should continue weaving together neuroscience, machine learning, and comparative studies, forging comprehensive models of how the brain implements RL across systems, scales, and timescales.

